# Prevalence of FMS Diagnosis According to ACR 2016 Revised Criteria in a Pain Therapy Centre in Italy: Observational Study

**DOI:** 10.3390/medicina60040599

**Published:** 2024-04-04

**Authors:** Vittorio Schweiger, Alvise Martini, Marta Nizzero, Eleonora Bonora, Giovanna Del Balzo, Leonardo Gottin, Lorena Torroni, Luca Polati, Giada Zuliani, Erica Secchettin, Enrico Polati

**Affiliations:** 1 Anesthesiology, Intensive Care and Pain Therapy Centre, Department of Surgery, Dentistry and Maternal Sciences, University of Verona, 37124 Verona, Italy; alvise.martini@univr.it (A.M.); marta.nizzero@gmail.com (M.N.); eleonora.bonora@aovr.veneto.it (E.B.); leonardo.gottin@univr.it (L.G.); pola.luca@hotmail.it (L.P.); giada.zuliani@studenti.univr.it (G.Z.); erica.secchettin@univr.it (E.S.); enrico.polati@univr.it (E.P.); 2 Department of Diagnostics and Public Health, Section of Forensic Medicine, University of Verona, 37134 Verona, Italy; giovanna.delbalzo@univr.it; 3 Department of Diagnostics and Public Health, Unit of Epidemiology and Medical Statistics, University of Verona, 37134 Verona, Italy; lorena.torroni@univr.it

**Keywords:** fibromyalgia, diagnostic criteria, prevalence

## Abstract

*Background and Objectives:* Fibromyalgia syndrome (FMS) is a multifaceted disease with a strong preference for the female sex. It is characterised by chronic widespread pain, sleep–wake disorders, fatigue, cognitive disturbances, and several other somatic symptoms. *Materials and Methods:* In this prospective observational study, we analysed data regarding 302 patients who were referred to our pain centre for a first clinical assessment evaluation and were then inspected for the physician-based 2016 revision of the ACR diagnostic criteria for FMS, regardless of the final diagnosis previously made by the pain therapist. *Results:* Among the 280 patients who adhered to the 2016 ACR questionnaire, 20.3% displayed positive criteria for FMS diagnosis. The level of agreement between the FMS discharge diagnosis made by the pain clinician and the ACR 2016 criteria-positivity was moderate (kappa = 0.599, with moderate agreement set at a kappa value of 0.6). Only four patients (1.7%) diagnosed as suffering from FMS at discharge did not satisfy the minimal 2016 ACR diagnostic criteria. *Conclusions*: This prospective observational study confirmed the diagnostic challenge with FMS, as demonstrated by the moderate grade of agreement between the FMS diagnosis at discharge and the positivity for 2016 ACR criteria. In our opinion, the use of widely accepted diagnostic guidelines should be implemented in clinical scenarios and should become a common language among clinicians who evaluate and treat patients reporting widespread pain and FMS-suggestive symptoms. Further methodologically stronger studies will be necessary to validate our observation.

## 1. Introduction

Fibromyalgia syndrome (FMS) is a multifaceted disease with a strong preference for the female sex (F:M ratio 4:1), characterised by chronic widespread pain, sleep–wake disorders, fatigue, cognitive disturbances, and several other somatic symptoms [1]. The FMS pathophysiology has not yet been fully elucidated, but several observations suggest its multifactorial nature, with different subsiding mechanisms and predisposing factors [2]. To date, FMS diagnosis lacks instrumental and/or laboratory pathognomonic markers and remains purely clinical. For research and clinical purposes, FMS diagnosis is now performed according to the latest 2016 American College of Rheumatology (ACR) revised criteria, while other diagnostic criteria were recently proposed [3,4]. According to observations inferred from the general population of different countries, FMS has an estimated global prevalence of 2 to 3% [5]. This prevalence is probably underestimated due to the presence of patients with low-intensity symptoms who might never even seek medical attention for the matter [6]. Moreover, there is uncertainty about the prevalence of FMS in patients referred to any pain therapy centre, where performing an FMS diagnosis is often challenging for several reasons [7]. Firstly, the patient and/or the clinician might focus more on localised pain, often forgetting or underestimating the underlying widespread pain and the typically associated symptoms. Furthermore, as demonstrated by the literature, FMS often overlaps or arises from other diseases, configuring the so-called concomitant or secondary FMS [8]. Moreover, there is still a sparse use of accepted diagnostic criteria in the clinical setting, and whether the patient suffers from FMS is often established by the clinician based on subjective or experiential criteria or sometimes on the inability to make a more accurate pain diagnosis [9]. Finally, many pain clinicians worldwide might still not recognise FMS as a dignified clinical entity and might choose to attribute the FMS-related symptoms to various psychiatric disorders [10]. All considered, FMS alone or in combination with other pain features (radicular pain, polyarthritis, headache, chronic low back pain, facial pain, or others) in a patient referring to a pain therapy centre will challenge pain clinicians not only for diagnosis but also for the uncertainty in the effectiveness of several pain treatment options, in particular with regards to invasive procedures [11,12]. This prospective observational study aimed to evaluate the prevalence of FMS in a population of chronic pain patients referring to a pain therapy centre for a first consultation, regardless of the motivation for which this evaluation was requested. Moreover, the agreement between entry and final diagnosis made by the pain clinician, particularly related to the presence of an FMS diagnosis, was analysed. The presence of concomitant diseases in patients with 2016 ACR-positive criteria for FMS was also evaluated.

## 2. Materials and Methods

### 2.1. Study Design

In this prospective observational study, we included male and female patients, aged ≥18 years, referring to our pain therapy centre for a first chronic pain evaluation regardless of the type of pain reported on admittance. At the end of the assessment, after the pain clinician made the final diagnosis, all the patients outside of the consultation room were requested by an independent physician (read not involved in the process of care) to respond to the 2016 ACR revised diagnostic criteria questionnaire for FMS (WPI [Widespread Pain Index] ≥ 7 and SSS [Symptom Severity Score] ≥ 5 or WPI 4–6 and SSS ≥ 9; pain in 4/5 bodily regions; symptoms at least ≥3 months; FMS diagnosis irrespective of other pain diagnoses). Patients who did not give informed consent to perform the questionnaire or came to the pain centre for a check-up were excluded from the study.

### 2.2. Endpoints

The primary endpoint was the prevalence of FMS in the evaluated population according to the 2016 ACR revised criteria. The secondary endpoint was the level of agreement between entry and final diagnoses made by the pain clinician, with a specific focus on the presence/absence of an FMS diagnosis. The presence of concomitant diseases in patients with ACR-positive criteria for FMS was also evaluated.

### 2.3. Ethics

All the study procedures were under the Helsinki Declaration of 1975/83. The study was approved by the local ethical committee (RED Register, 1751CESC).

### 2.4. Data Collection and Statistical Analysis

We collected demographic, medical, and clinical data of the evaluated patients at the time of the 2016 ACR revised criteria questionnaire administration. We also recorded the family physician indication for a first specialist pain evaluation (i.e., entry diagnosis) and the final diagnosis made by our pain clinician. All data were entered into a paper CRF (case report form) and then transferred to an electronic database. Descriptive statistics summarised demographic, medical, and clinical characteristics. The categorical variables were expressed as numbers and percentages, while quantitative variables were expressed as medians and interquartile ranges (I-III quartile). The level of concordance between the entry and final diagnosis was analysed using Cohen’s kappa interrater agreement measure, which calculates the agreement between judgements expressed by different operators. The kappa statistic measure of agreement is scaled to 0 when the amount of agreement is expected to be observed by chance and 1 when there is perfect agreement. The following interpretations are suggested for intermediate agreement values: values <0.40 indicate a slight/poor, between 0.40 and 0.79 fair/moderate, and >0.80 indicate a substantial/almost perfect agreement.

## 3. Results

### 3.1. Study Population

Between March and September 2021, 302 patients (202 females, 66.8%; and 100 males, 33.1%) were referred to our pain therapy centre for the first time and were evaluated by different pain clinicians. Of the initial cohort, 22 patients (7.2%) did not consent to the study and were excluded from the analysis. Patient demographics and characteristics are summarised in Table 1.

### 3.2. Primary Endpoint

Out of 280 patients, applying the 2016 ACR diagnostic criteria showed an FMS prevalence of 20.3% (57 patients). The demographics and other characteristics of this population are summarised in Table 2.

### 3.3. Secondary Endpoints

**Figure 1:**
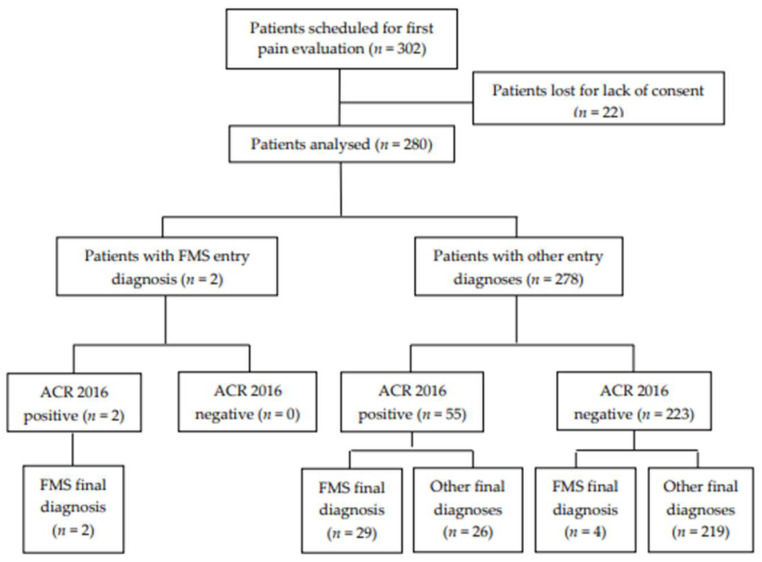
Flowchart of the correspondence of the study population. FMS = fibromyalgia syndrome; ACR 2016 = American College of Rheumatology Revised Criteria for FMS diagnosis.

In the 57 patients with positive ACR criteria 2016, a final diagnosis of FMS was made by the pain clinicians in 31 patients (54.5%), while 26 patients (45.6%) were not diagnosed with FMS (Figure 1). In particular, in the two patients with FMS entry diagnosis, a concordant FMS diagnosis was performed at discharge. Both patients displayed positive 2016 ACR criteria for FMS. Regarding the other 55 patients with positive ACR criteria, the FMS final diagnosis was made in 29 patients (52.7%). In comparison, 26 patients (47.2%) were diagnosed differently at discharge (Table 3). Moreover, in the 223 patients with negative ACR criteria, a final FMS diagnosis was only made in four cases (1.7%), whereas a different discharge pain diagnosis was confirmed in 219 patients (Table 4).

Out of 31 patients with positive 2016 ACR criteria and an FMS discharge diagnosis, a primary FMS was identified in 19 patients (61.2%), while in 12 patients (38.7%) the FMS was considered as concomitant with other diseases like rheumatological diseases (8 patients), neurological diseases (2 patients), osteoarthritis (1 patient), and others (1 patient). In the four patients with negative 2016 ACR criteria and an FMS discharge diagnosis, a primary FMS was identified in three patients (1.7%). In one of these patients, the FMS was considered concomitant with a rheumatological disease. The level of agreement in the FMS diagnosis made by pain clinicians at the end of the examination and the results from the 2016 ACR criteria for FMS outside the examination room was 0.599. According to Cohen’s kappa calculation, this value indicates a moderate agreement between the final diagnosis made by pain clinicians and the resulting scores according to the ACR 2016 diagnostic criteria.

## 4. Discussion

The prevalence of FMS in the general population is still debated and varies among countries, with an estimated range between 2 and 8% [13]. This variability is related to several factors, such as differences in diagnostic criteria, studies’ methodologies, and populations [14]. Moreover, the prevalence of FMS among pain centres has not been fully established, reflecting some concerns by pain physicians regarding FMS diagnosis, like the sparse use of recommended diagnostic criteria, the recognition of FMS as a real clinical entity, the prevalent focus on localised or district pain, and the preference of some clinicians for specific diagnoses and related pain treatments. Furthermore, the failure to perceive the presence of warning signs like the history of pain in childhood and adolescence, the onset of widespread pain after physical and/or psychosocial stress, and the presence of multiple gastrointestinal, urological, gynaecological, and neurological somatic symptoms may prevent or delay a correct FMS diagnosis and subject the patient to inappropriate or useless treatments [6]. In this study, of 302 patients referring to our pain centre for a first evaluation for chronic pain, only 2 patients, i.e., 0.6% of the total, were referred to our centre for FMS. Meanwhile, another 32 patients (10.6%) were admitted with a history of generic widespread pain with no previous mention of FMS. The majority of these patients had a diagnosis on the admission of LBP, headache or facial pain, cervical or neck pain, articular pain, and others. The small number of FMS diagnoses on admission may be related to some concerns, like the poor knowledge among physicians about FMS or real scepticism in the medical community regarding this syndrome and its related conditions [15,16,17]. Nevertheless, the systematic application of the 2016 ACR revised diagnostic criteria for FMS to these patients after the clinical evaluation outside the examination room highlighted a prevalence of positive criteria for FMS in 57 patients (20.3%). In the literature, the prevalence of FMS according to different diagnostic criteria was reported mostly by rheumatologists upon clinical evaluation and was estimated to range from 10 to 15% of evaluated patients [18,19]. Nevertheless, very few studies investigated the prevalence of FMS in patients referring to pain centres or other non-rheumatological institutions. Lee and colleagues, using the modified 2010 ACR criteria on 1233 patients referring to a tertiary pain centre, reported an FMS prevalence of 11%, with the exclusion of patients who had previously been diagnosed with FMS [7]. An Italian observation on 151 patients referring to an academic podiatry clinic where the Italian version of the Fibromyalgia Survey Diagnostic Criteria (FSDC) was used as the main diagnostic tool, revealed a prevalence of FMS of 13.9%, thus confirming the importance of the syndrome in this selected population [20]. Regarding the diagnostic concordance between the 2016 ACR-positive criteria for FMS and the discharge FMS diagnosis made by pain clinicians, our data showed a kappa value = 0.599, reflecting a moderate level of agreement according to the theoretical model [21]. Indeed, of over 55 patients who satisfied diagnostic criteria and were referred to the centre without the “warning sign” of an FMS diagnosis on admission, 26 patients (47.2%) did not receive an FMS diagnosis, nor was FMS present as a concomitant diagnosis in the final documentation. In addition, 17 of these patients (30.9%) were diagnosed with pure localised pain, suggesting the pain clinician or patient’s focus on specific bodily regions, particularly the head and lower back. These data suggest an evenly unsatisfactory accuracy in the FMS diagnostic approach, both in general and in an academic setting with a specific expertise on pain therapy, confirmed by other observations in the literature. In a similar study, Wolfe and colleagues reported that in a rheumatology university clinic, among 121 FMS patients, identified with the 2011 ACR criteria, modified for self-report from the 2010 ACR preliminary diagnostic criteria, clinicians failed to identify 60 criteria-positive patients (49.6%) and incorrectly identified as fibromyalgic 43 criteria-negative patients (11.4%). The diagnostic agreement beyond chance was reported by the authors as fair (kappa = 0.41) [22]. Several reasons may lead to FMS misdiagnosis. In a recent worldwide survey aimed at investigating whether medical doctors are familiar with the use of ACR criteria for FMS diagnosis, only 10% of these clinicians adhered to the ACR criteria and FMS diagnosis was mostly based on the presence of generic widespread pain, unrefreshed sleep, fatigue, and cognitive problems [9]. This is probably due to the perception of ACR criteria as a pure research tool, hence not useful for clinical purposes. Instead, the physician-based ACR criteria demonstrated their validity in the clinical setting for individual FMS diagnosis with reportedly good sensitivity and specificity [3]. Furthermore, many clinicians, also in the pain centres context, doubt FMS as a real pathologic entity and believe that these patients suffer from other clinical conditions such as a subsiding untreated depression, persistent somatoform pain disorder, brain disease, or neuropathic pain from small fibres neuropathy (SFN) [10]. Many clinicians avoid diagnosing FMS by believing that this may be unhelpful for patients due to some otherwise potential negative implications of the FMS label on patients’ state of depression, anxiety, and proneness to catastrophizing. On the contrary, all recent guidelines recommend that FMS diagnosis should be communicated to patients after the initial evaluation to reduce anxiety, repeated unnecessary diagnostic procedures, and inappropriate drug treatments [22]. Finally, some clinicians might underestimate the components of widespread pain and associated symptoms in patients with predominant localised pain like migraine, craniofacial pain, chronic pelvic pain, or other regional pain problems that may overlap each other and blur the clinical picture [2]. On the patient’s side, an FMS label may affect personal credibility and dignity, having them struggle to convince physicians that their illness is not imaginary or psychological. Hence, some patients will likely be reluctant to refer to the physician about either a previous FMS diagnosis or the widespread pain and associated symptoms, highlighting strictly localised pain to avoid stigmatisation from health care providers [23].

## 5. Study Limitations

Besides the strength of the diagnostic accuracy, the study shows some limitations. In particular, the specifics of how the single physician conducted the clinical evaluation and whether they applied the 2016 ACR diagnostic criteria were not investigated. It was, therefore, impossible to compare the diagnostic efficiency of the clinician with and without the use of the ACR guidelines. Additionally, the different levels of expertise of the pain clinicians performing the single evaluation might affect the accuracy of the final pain diagnosis.

## 6. Conclusions

This prospective observation confirmed the relevant prevalence of FMS in a pain therapy centre and also the difficulty for the clinicians in diagnosing FMS, as demonstrated by the moderate level of agreement between the FMS diagnosis on discharge and the 2016 ACR criteria-positivity. In our opinion, the use of accepted diagnostic criteria must be implemented in the clinical scenarios, thus hopefully becoming a widely endorsed language among clinicians who evaluate and treat patients reporting widespread pain and FMS-suggestive associated symptoms. Further methodologically stronger studies will be necessary to validate our observation.

## Figures and Tables

**Table 1:** Demographics and baseline characteristics of evaluated population (302 patients).

Patients Evaluated, *n*	302
Gender, *n* (%)	
Female	202 (66.8%)
Male	100 (33.1%)
Age, yr. *	61 (51–75)
Entry diagnosis, *n* (%)	
Low back pain	126 (41.7%)
Headache or facial pain	40 (13.2%)
Chronic widespread pain	32 (10.6%)
Cervical or neck pain	28 (9.2%)
Articular pain	25 (8.2%)
Dorsal pain	13 (4.3%)
Abdominal pain	11 (3.6%)
Vascular pain	10 (3.3%)
Neuropathic pain	8 (2.6%)
Pelvic pain	7 (2.3%)
Fibromyalgia	2 (0.6%)

* = Median (IQR).

**Table 2:** Demographics and clinical characteristics of the patients responding as positive to ACR 2016 diagnostic criteria for FMS (57 patients).

Patients with Positive ACR Criteria, *n*	57
Gender, *n* (%)	
Female	53 (92.9%)
Male	4 (7.02%)
F:M ratio	14:1
Age, yr. *	57 (43–66)
Entry diagnosis, *n* (%)	
Chronic widespread pain	22 (38.6%)
Low back pain	12 (21%)
Headache or facial pain	7 (12.2%)
Articular pain	5 (8.7%)
Cervical or neck pain	4 (7%)
Dorsal pain	3 (5.2%)
Fibromyalgia	2 (3.5%)
Neuropathic pain	2 (3.5%)
ACR 2016 items *	
WPI (0–19)	13 (10–15)
SS (0–12)	9 (6.5–10)
VAS (0–100) *	80 (70–90)

* = Median (IQR): WPI = Widespread Pain Index; SS = Symptom Severity Score; VAS = Visual Analogue Scale.

**Table 3:** Final diagnosis in ACR-positive patients (57 patients).

Patients with Positive ACR Criteria, *n*	57
Final diagnosis, *n* (%)	
FMS	31 (54.5%)
Primary FMS	19 (61.2%)
Concomitant FMS	12 (38.7%)
WP from rheumatic disease	6 (10.5%)
WP from ostheoarthritis	5 (8.7%)
Neuropathic pain	4 (7%)
Migraine	4 (7%)
Mixed pain on upper limbs	3 (5.2%)
LBP from vertebral fracture	2 (3.5%)
Sacroiliac joint pain	2 (3.5%)
Knee pain	1 (1.7%)
Tension-type headache (TTH)	1 (1.7%)

FMS = fibromyalgia syndrome; WP = widespread pain; LBP = low back pain.

**Table 4:** Final diagnosis in ACR-negative patients (223 patients).

Patients with Negative ACR Criteria, *n*	223
Final diagnosis, *n* (%)	
LBP	91 (40.8%)
Headache	26 (11.6%)
Cervical pain	18 (8%)
Chronic abdominal pain	12 (5.3%)
Dorsal pain	11 (4.9%)
Facial pain	10 (4.4%)
Pain from ostheoarthritis	9 (4%)
Vascular pain on lower limbs	8 (3.5)
Cancer pain	8 (3.5)
Neuropathic pain	8 (3.5)
Scapular pain	6 (2.6%)
Coccigodynia	5 (2.2%)
FMS	4 (1.7%)
Primary FMS	3 (75%)
Concomitant FMS	1 (25%)
WP (unspecified)	3 (1.3%)
Thoracic pain	2 (0.9%)
Anal pain	2 (0.9%)

FMS = fibromyalgia syndrome; WP = widespread pain; LBP = low back pain.

## Data Availability

The raw data supporting the conclusions of this article will be made available by the authors on request.
